# High Intensity Physician-Based Service Use for Mental Health Concerns in a General-Population Sample of Children and Youth: Utilisation des services de haute intensité dispensés par des médecins pour les problèmes de santé mentale dans un échantillon d’enfants et de jeunes de la population générale

**DOI:** 10.1177/07067437241300961

**Published:** 2025-01-06

**Authors:** Jordan Edwards, Li Wang, Anne E. Fuller, Kelly K. Anderson, Claire de Oliveira, Katholiki Georgiades

**Affiliations:** 1 Department of Psychiatry and Behavioural Neurosciences, 3710 McMaster University, Hamilton, ON, Canada; 2 Offord Centre for Child Studies, 3710 McMaster University, Hamilton, ON, Canada; 3 Department of Pediatrics, 3710 McMaster University, Hamilton, ON, Canada; 4 Department of Epidemiology & Biostatistics, Western University, London, ON, Canada; 5 Department of Psychiatry, Schulich School of Medicine, Western University, London, ON, Canada; 6 Lawson Health Research Institute, London, ON, Canada; 7 Institute of Health Policy, Management and Evaluation, University of Toronto, Toronto, ON, Canada; 8 7978 Centre for Addition and Mental Health, Institute for Mental Health Policy Research and Campbell Family Mental Health Research Institute, Toronto, ON, Canada; 9 Centre for Health Economics and Hull York Medical School, University of York, York, UK

**Keywords:** child & youth, mental health services, data linkage, high-intensity service use

## Abstract

**Objective:**

To examine factors associated with high intensity physician-based mental health care services in a population-based sample of children and youth in Ontario, Canada.

**Methods:**

Data from the 2014 Ontario Child Health Study (OCHS) were linked at the person-level to longitudinal health administrative databases containing physician contacts in outpatient settings, emergency departments and hospitals. Our analytical sample (15.8% of 9,301, *n* = 1,423) included children and youth with at least one physician-based contact for a mental health concern in the 24-month period post-OCHS. Over the same follow-up period, we classified high intensity service use as those in the top 10th and fifth percentiles of physician-based mental health service cost contributors. Costs were assessed using physician billing data, as well as estimated emergency department visit and hospitalization costs.

**Results:**

Among those with at least one contact, being older (PR: 1.15, 95% CI: 1.04, 1.25), having more severe symptoms of mental ill-health (PR: 1.04, 95% CI: 1.01, 1.06) and having a history of mental health service use (PR: 3.99, 95% CI: 1.37, 11.61), were positively associated with high-intensity service use, while living in a rural setting (PR: 0.35, 95% CI: 0.15, 0.30) was negatively associated. Findings were largely consistent between the top 10th and fifth percentiles. Notably, among youth ages 14–17 years, self-reported prior suicide attempt was positively associated with high-intensity (top fifth percentile) service use (PR: 6.09, 95% CI: 1.41, 26.26).

**Conclusions:**

Our findings suggest older age, non-rural residency, mental health symptom severity and suicidal behaviour are important factors associated with high-intensity physician-based mental health service use. Our findings will inform efforts to better identify children and youth who may benefit from early and personalized interventions.

## Plain Language Summary Title

Understanding Children and Youth with the Greatest Mental Health Related Service Needs.

Plain Language Summary

Why was the study done? There is a need to better understand children and youth who have the greatest mental health related service needs. Existing data gaps have limited our ability to identify important factors associated with children and youth with relatively high amounts of mental health service use. Our goal was to fill this knowledge gap by making use of multiple data sources, each capturing different information, that have been linked together. What did the researchers do? Data from the 2014 Ontario Child Health Study, a survey of 10,802 children and youth aged 4-17 years across Ontario, Canada, were linked at the person-level to health service records capturing information on all physicians, emergency departments, and hospitals, across the province. We identified children and youth with high service needs as those in the top 10th and fifth percentiles of costs related to service use in the 2-year period following completion of the survey. Costs were assessed using physician billing data, as well as estimated emergency department visit and hospitalization costs. What did the researchers find? We found that being older, having more severe symptoms of mental illness, and having a history of prior mental health service use were all positively associated with being identified in a high service needs group. We found that living in a rural area was negatively associated with being identified as having high service needs. We also found that among youth ages 14-17, self-reported suicide attempt was positively associated with being in a high service needs group. What do the findings mean? Our findings suggest older age, non-rural residency, mental health symptom severity, and suicidal behaviour are important factors associated with children and youth with the greatest mental health related service needs. This work will inform efforts to better identify children and youth who may benefit from early and personalized interventions.

## Résumé

**Objectif:**

examiner les facteurs associés aux services de soins de santé mentale de haute intensité dispensés par des médecins dans un échantillon d’enfants et de jeunes en Ontario, au Canada.

**Méthodologie:**

les données de l’Enquête sur la santé des jeunes Ontariens (ESJO) 2014 ont été reliées au niveau des individus à des bases de données administratives longitudinales sur la santé répertoriant les contacts avec les médecins dans les services ambulatoires, les services d’urgence et les hôpitaux. L’échantillon analysé (15,8 % de 9 301, n = 1 423) comprenait des enfants et des adolescents ayant eu au moins un contact avec un médecin pour un problème de santé mentale au cours de la période de 24 mois suivant l’ESJO. Au cours de la même période de suivi, nous avons classé l’utilisation des services de haute intensité dans les 10^e^ et 5^e^ centiles supérieurs des éléments de coût liés aux services de santé mentale fournis par les médecins. Les coûts ont été évalués à partir des données de facturation des médecins, ainsi que des coûts estimés des visites aux urgences et des hospitalisations.

**Résultats:**

parmi les personnes ayant eu au moins un contact, le fait d’être plus âgé (RP : 1,15, IC : 95 % 1,00 à 1,25), d’avoir des symptômes de mauvaise santé mentale plus graves (RP:1,04, IC : 95 % 1,01 à 1,06) et d’avoir déjà utilisé des services de santé mentale (RP : 3,99, IC : 95 % 1,37 à 11,61) était positivement associé à l’utilisation de services de haute intensité, tandis que le fait de vivre en milieu rural (RP : 0,35, IC : 95 % 0,15 à 0,30) était négativement associé à l’utilisation de services de haute intensité. Les résultats étaient globalement cohérents entre les 10^e^ et 5^e^ centiles supérieurs. Notamment, chez les jeunes âgés de 14 à 17 ans, une tentative de suicide antérieure autodéclarée était positivement associée à l’utilisation de services de haute intensité (5^e^ centile supérieur) (RP : 6,09, IC : 95 % 1,41 à 26,26).

**Conclusions:**

nos résultats laissent penser que le fait d’être plus âgé, de résider dans une zone non rurale, de présenter des symptômes de trouble mental graves et d’avoir un comportement suicidaire sont des facteurs importants associés à l’utilisation de services de santé mentale à haute intensité fournis par un médecin. Nos résultats contribueront à mieux reconnaître les enfants et les jeunes susceptibles de bénéficier d’interventions précoces et personnalisées.

## Introduction

Approximately one in five Canadian children and youth have a mental disorder at any given time.^1^ Across all age groups, mental disorders are estimated to be the fourth leading cause of health expenditures in Canada, with an estimated direct cost of $13.1 billion in 2010.^2^ Between the years of 2017 and 2018, community mental health and addictions services across Canada accounted for nearly a fifth of total public health expenditures at $1.9 billion.^3^ Among children and youth ages birth-14 years, mental disorders consistently represent one of the top five contributors to costs from physicians, hospitals and drug expenditures.^2^ These trends are similar in other settings. For example, evidence from the US suggests that mental disorders account for the largest proportion of healthcare spending among children and youth, compared to other disorders.^4^

Over the past decade, the prevalence of mental ill health has been increasing among youth in Canada and globally.^5–7^ In Canada, the largest increases have been observed for symptoms of depression, anxiety and suicidality, which have been shown to disproportionately impact females to a greater extent than males.^6^ Perceptions of need for professional help for mental health-related concerns have nearly tripled among children and youth ages 4–16 years in Ontario between 1983 and 2014.^8^ This finding aligns with evidence of increases in service utilization for mental ill-health among youth in Ontario, with the greatest increases seen in acute care settings.^9^ Although the overall rate of service use among children and youth for mental health concerns has increased, the distribution of service use across population subgroups remains uneven.^10^

To date, evidence has focused on identifying and characterizing children and youth who are underserved, which has important implications for service planning and the development of targeted outreach services.^10^ However, studying children and youth who use the greatest amount of healthcare resources is also important to inform service planning and targeting.^11^ High-intensity service use represents a period of intense intervention that may involve care from various providers aiming to support people with significant functional impairment and or risk factors related to severe mental illness.^12^ There is currently little research identifying factors associated with relative high-intensity use of physician-based outpatient and acute care services for mental health concerns among children and youth, both in Canada and internationally.^11^ This may be due, in part, to the limited availability of data required to conduct this research, including the need for detailed socio-demographic and clinical factors, in combination with longitudinal service use records. This is an important evidence gap, as children, youth and their families who use a greater intensity of physician-based mental health services may benefit from personalized intensive clinical programs aimed at improving health and functioning and reducing the burden of suffering.

Using population-based survey data of children and youth aged 4–17 years linked retrospectively and prospectively to health administrative data,^10,13^ our objective was to examine factors associated with high-intensity mental health-related physician-based outpatient, emergency and hospital care contacts, estimated as those in the top 10th and fifth percentile of mental health related cost distributions in the 24-month period following completion of the survey. The goal of this work is to support policy and decision makers as they plan and develop targeted mental health services by improving our understanding of children and youth with the greatest mental health-related service needs.

## Methods

### Study Design

Data for the current analyses comes from the 2014 Ontario Child Health Study (2014 OCHS), a cross-sectional epidemiologic survey of 10,802 children and youth aged 4–17 years (50.8% response rate) in Ontario, Canada. The sampling frame for the 2014 OCHS was the Canadian Child Tax Benefit (CCTB) file, with coverage of over 95% of households with children and youth aged 4–17 years in Ontario.^13^ Households from the CCTB were selected using a stratified, clustered and random sampling approach.^13^ Further details regarding study design can be found elsewhere.^13^

### Data Linkage and Analytic Sample

**Figure 1.**
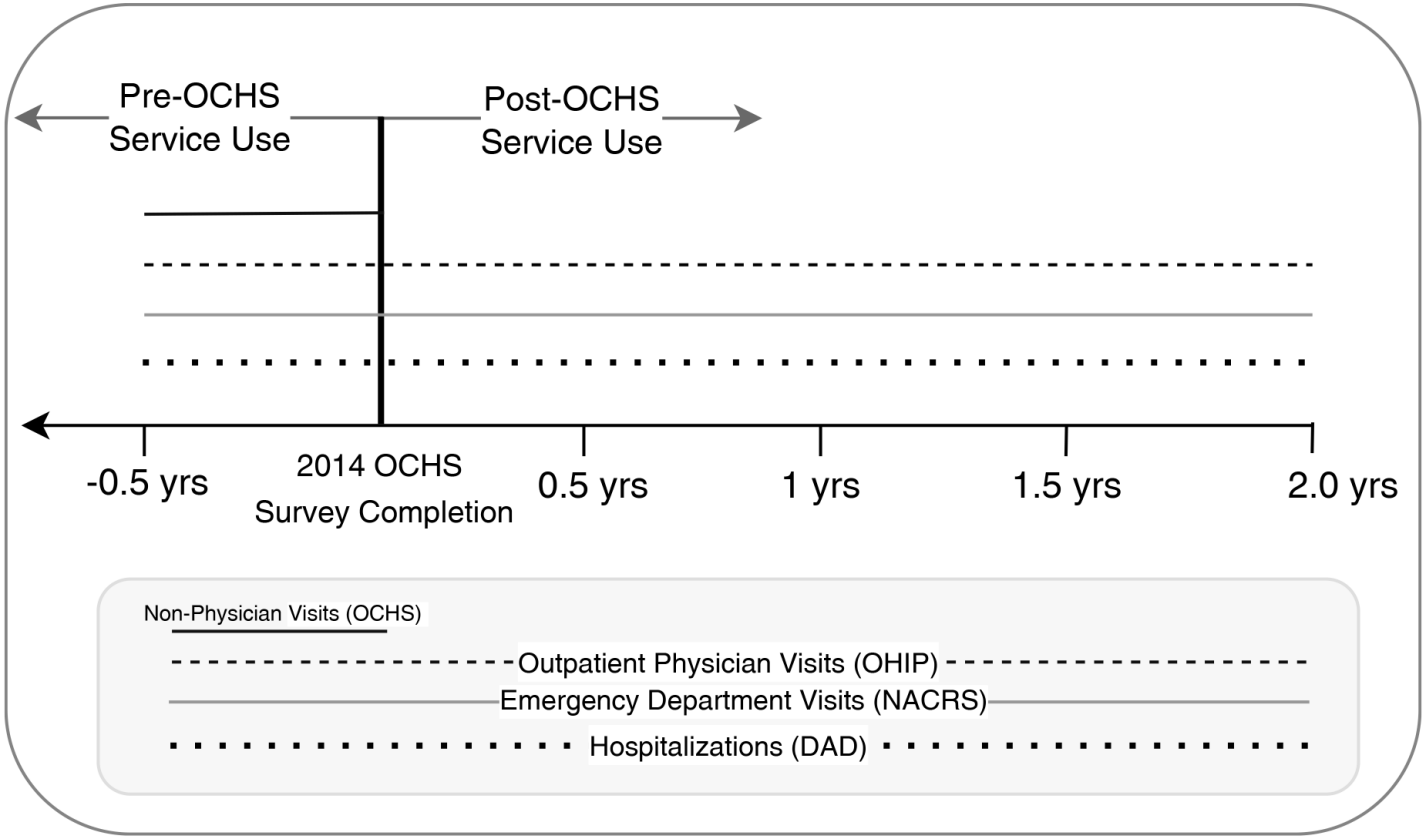
Study timeline and data linkage.

The eligible sample for analyses in the present study included OCHS participants who agreed to share their data with the Ministry of Health and Long-term Care in Ontario (*n* = 9,666, 89.5% of total) and whose data were successfully linked to retrospective and prospective health administrative databases (*n* = 9,301, 96.2% of those who agreed). Individually linked health administrative data provided information on all medically necessary physician services covered under the Ontario Health Insurance Plan (OHIP).^14^ This included information from outpatient physician services, including visits to family physicians, pediatricians and psychiatrists. Data from hospitalizations were obtained from the Discharge Abstract Database (DAD), and emergency department visits were obtained from the National Ambulatory Reporting System (NACRS). A full data dictionary, including a list and description of linked databases, can be found in Supplement 1. This work followed the RECORD guidelines (see Supplement 2).^15^ Figure 1 outlines the timeline of the study and a chart of databases used.

### Primary Outcome Measure—Physician-Based Mental Health Related Service Costs in the 24-Month Period Following the OCHS Survey

Mental health-related service costs were obtained from health administrative data using the OHIP claims, NACRS and DAD databases. Mental health-related service contacts were identified using physician diagnostic codes assigned to each presentation (Supplement 1). Costs were estimated by multiplying physician OHIP fees for each presentation by the number of services used. For emergency department visits and hospitalizations, costs were estimated by multiplying setting specific Resource Intensity Weights (RIW)—an estimate of the relative resource use based on methodology that groups patients by diagnoses and adjusts for the presence of co-morbidities and intervention—by the Cost per Comprehensive Ambulatory Classification System Weighted Case (CPWC) for emergency department visits and Cost of a Standard Hospital Stay for hospitalizations, both of which were obtained from the Canadian Institute for Health Information.^16,17^

We assessed cumulative physician-based service use and associated costs over the 24-month period post-OCHS survey completion. Using cost data, we classified children and youth into high-versus low-intensity service use groups. We examined two cut-offs for high-intensity service use based on previous work looking at patterns of high service utilization.^18^: top 10th and top fifth percentile of cumulative cost distributions for all physician-based mental health-related service use. We did not examine the top 1 percentile due to small samples sizes. Note that there was no loss to follow-up in our sample from the date of completing the survey until the 24-month period post-OCHS.

### Other Measures

Data on socio-demographic, health-related measures and prior service-related factors, as well as parental mental health factors, were obtained from OCHS survey responses and retrospective administrative data. These included measures of child and youth (a) age; (b) sex assigned at birth; (c) number of biological parents in the home; (d) household income relative to Statistics Canada's low-income measure^19^; (e) parental immigrant background; and (f) rurality, which was derived from population density and size^20^; (g) mental health symptom ratings, which were obtained using the Ontario Child Health Study Emotional Behavioural Scales, which were converted to a summative scale of total symptoms where high was indicative of more severe symptoms^21,22^; (h) impairment related to mental health symptoms interfering with aspects of daily life; (i) childhood chronic condition status; (j) self-reported information on substance use including indicators related to smoking, binge drinking and drug use among youth ages 14–17; (k) self-reported information on prior suicide attempts among youth ages 14–17; (l) self-reported problematic eating among youth ages 14–17; (m) self-reported prior childhood abuse among youth ages 14–17; (n) mental health related service contacts prior to completing the OCHS estimated using a combination of administrative data to obtain physician services and self-reported contacts with non-physician services. We also included parental health factors including psychological distress using the K-10.^23^ Full details of our measures can be found in Supplement 1.

### Analysis

Socio-demographic, clinical and service-related factors, and their association with high-intensity physician-based service use, compared to moderate/low-intensity use were assessed. This analysis was conducted separately for those belonging to the top 10th percentile (compared to bottom 90th percentile) and top fifth percentile (compared to bottom 95th percentile) of cumulative cost distributions. Separate analyses were conducted for the total sample of children and youth ages 4–17 years who had at least one mental health-related service contact, and the sub-sample of youth ages 14–17 years who had additional information on self-reported clinical and behavioural factors. To increase the power of sub-sample analysis of youth ages 14–17 we included everyone in our analysis regardless of service contact, where those with no contact were allocated a cost of $0. These associations were modelled using modified Poisson regression analyses to estimate prevalence ratios (PR). Sample weights were used to generate representative estimates, and mean bootstrap weights were used to support complex survey design to create accurate standard errors.^13^ There was minimal missing data in our sample (<10%), as such, a complete case analysis was conducted. Across our analyses alpha was set to 0.05. All analyses were conducted using STATA (version 14).^24^

## Results

Our analytical sample included 1,423 children and youth who had at least one physician-based mental health-related service contact in the 24-month period following the OCHS survey date (1423/9301 = 15.8% of the linked sample). See Table 1 for sample characteristics of our total and analytical samples.

**Table 1.** Total Linked Sample and Analytical Sample Characteristics.

Outcomes	Total linked sample (*n* = 9,301) % (95% CI)	Analytical sample (*n* = 1,423) % (95% CI)	Analytical sample youth 14–17 (*n* = 466) % (95% CI)
Sex (male)	51.6 (50.6, 52.7)	58.9 (56.2, 61.5)	51.4 (46.5, 56.4)
Age (continuous)	10.6 (10.5, 10.7)	11.1 (10.9, 11.3)	15.6 (15.5, 15.7)
Household poverty (poor)	18.4 (17.6, 19.2)	19.3 (17.2, 21.4)	15.5 (11.9, 19.1)
Biological parents in home (one or no biological parents in the home)	19.5 (18.7, 20.3)	32.4 (29.9, 34.9)	42 (37.1, 46.9)
Parent immigration status (immigrant)	42.6 (41.6, 43.7)	32.4 (29.9, 34.9)	29.2 (24.7, 33.7)
Urban-rural residency (rural)	30.8 (29.8, 31.8)	25.1 (22.8, 27.4)	25.3 (21, 29.7)
OCHS-scales total score (continuous)	10.4 (10.2, 10.6)	17.1 (16.3, 17.8)	16.7 (15.2, 18.1)
Mental health-related impairment (continuous)	4.2 (4.1, 4.3)	5.4 (5.1, 5.6)	5.4 (4.9, 5.9)
Prior service use (yes)	21.7 (20.9, 22.6)	52.2 (49.5, 54.9)	48.1 (43.1, 53)
Presence of chronic condition (yes)	6.1 (5.6, 6.6)	10.5 (8.9, 12.2)	13.9 (10.5, 17.3)
Prior suicide attempt (yes)			14.5 (11, 17.9)
Prior childhood abuse (yes)			41.3 (36.4, 46.2)
Problematic eating (continuous)			4.2 (3.7, 4.6)
Smoking (yes)			21.6 (17.5, 25.7)
Binge drinking (yes)			11.8 (8.6, 15)
Prior drug use (yes)			18.6 (14.8, 22.5)

Among children and youth ages 4–17 years, being older (PR: 1.15, 95% CI: 1.04, 1.28), having higher levels of mental health symptoms (PR: 1.04, 95% CI: 1.01, 1.06), and having prior mental health-related service use in the 6-months pre-OCHS (PR: 3.99, 95% CI: 1.37, 11.61) were significantly associated with being identified in a high-intensity service use group (top 10th percentile of cumulative costs distribution) (See Table 2). Living in a rural setting, compared to a non-rural setting (PR: 0.35, 95% CI: 0.15, 0.30), was negatively associated with being identified in a high-intensity service use group (top 10th percentile) (See Table 2). Similar associations were found when examining the top fifth percentile. One note is that living in a rural setting was no longer significantly associated with being in the top fifth percentile.

**Table 2.** Factors associated with children and youth with future high intensity (top 10th and fifth percentile of cumulative cost distributions) service use. Stratified findings from youth ages 14–17 to assess more detailed youth reported mental health and substance use outcomes.

Outcomes	Total sample ages 4–17		Youth ages 14–17	
Variable	Being in the top 10th percentile (*n* = 142) PR (95% CI)	Being in the top fifth percentile (*n* = 71), PR (95% CI)	Being in the top 10th percentile (*n* = 70), PR (95% CI)	Being in the top fifth percentile (*n* = 40), PR (95% CI)
Sex				
Female	Ref	Ref	Ref	Ref
Male	1.3 (0.67, 2.49)	0.8 (0.35, 1.83)	2.18 (0.82, 5.76)	0.81 (0.28, 2.39)
Age (continuous)	1.15* (1.04, 1.28)	1.2* (1.09, 1.33)	0.76 (0.48, 1.19)	0.75 (0.42, 1.33)
Household poverty				
Not poor	Ref	Ref	Ref	Ref
Poor	0.86 (0.41, 1.81)	2.26 (0.95, 5.37)	0.6 (0.21, 1.7)	1.24 (0.27, 5.58)
Number of biological parents in home				
Two	Ref	Ref	Ref	Ref
One or no biological parents	1.49 (0.63, 3.53)	1.1 (0.44, 2.76)	0.71 (0.21, 2.45)	1.23 (0.32, 4.81)
Parent immigration status				
Non-immigrant	Ref	Ref	Ref	Ref
Immigrant	0.78 (0.37, 1.64)	0.49 (0.14, 1.69)	0.61 (0.23, 1.63)	0.28 (0.04, 1.85)
Urban–rural residency				
Non-rural	Ref	Ref	Ref	Ref
Rural	0.35* (0.15, 0.8)	0.91 (0.33, 2.57)	0.22* (0.07, 0.66)	0.35 (0.08, 1.51)
OCHS-scales				
Total score (continuous)	1.04* (1.01, 1.06)	1.04* (1.01, 1.06)	1.01 (0.97, 1.06)	0.99 (0.94, 1.04)
Mental health-related impairment				
Total score (continous)	1.02 (0.96, 1.09)	1.01 (0.92, 1.12)	1.04 (0.93, 1.16)	1.09 (0.92, 1.29)
Prior service use				
No	Ref	Ref	Ref	Ref
Yes	3.99* (1.37, 11.61)	5.17* (1.82, 14.68)	7.4*(1.87, 29.27)	4.4* (1.12, 17.21)
Presence of chronic condition				
No	Ref	Ref	Ref	Ref
Yes	1.17 (0.39, 3.5)	1.31 (0.38, 4.56)	0.77 (0.15, 3.89)	0.78 (0.16, 3.79)
Parental distress (continuous)	1.02 (0.95, 1.1)	0.97 (0.9, 1.04)	–	–
Parental mental health diagnosis				
No	Ref	Ref	-	-
Yes	1.74 (0.78, 3.91)	0.64 (0.26, 1.58)		
Prior suicide attempt	–	**–**		
No			Ref	Ref
Yes			3.34 (0.9, 12.33)	6.09* (1.41, 26.26)
Prior childhood abuse	–	**–**		
No			Ref	Ref
Yes			2.34 (0.88, 6.21)	0.88 (0.21, 3.71)
Problematic rating	–	**–**		
Total score (continuous)			1.04 (0.94, 1.15)	1.1 (0.98, 1.23)
Smoking	–	**–**		
No			Ref	Ref
Yes			1.12 (0.28, 4.52)	0.4 (0.09, 1.81)
Binge drinking	–	**–**		
No			Ref	Ref
Yes			0.56 (0.15, 2.06)	1.96 (0.62, 6.21)
Prior drug use	–	**–**		
No			Ref	Ref
Yes			1.11 (0.34, 3.66)	2.34 (0.66, 8.31)

The use of “*” and bold text indicates a significant association, as the confidence interval for the prevalence ratio does not pass through 1.

Among youth ages 14–17 years, having prior mental health-related service use was significantly associated with high intensity service use (10th percentile) (PR: 7.40, 95% CI: 1.87, 29.3). Living in a rural setting, compared to a non-rural setting, was negatively associated with future high-intensity service use (10th percentile) (PR: 0.22, 95% CI: 0.07, 0.66). When assessing the top fifth percentile, having a history of prior service use remained significantly associated, whereas the association with living in a rural setting became non-significant. There was, however, a significant association between past 12-month suicide attempt and high-intensity service use (fifth percentile) (PR: 6.09, 95% CI: 1.41, 26.26). Full details on these associations are available in Table 2.

## Discussion

Our findings suggest that roughly one in seven children and youth received physician-based mental health care in the 2-year period following completion of the OCHS survey. Among these children and youth, we found distinct socio-demographic and clinical characteristics associated with high-intensity physician-based mental health service use. We found an association between prior mental health service use and older age and high-intensity service use, as well as an inverse association between rural residence and high-intensity service use. These findings are consistent with our understanding of two concepts related to mental health care access and age of onset of mental disorders.^25–27^ Mainly, it is well established that one of the best predictors of future mental health service use is prior mental health service use and that the peak age of onset of mental disorders is in adolescence.^25,28,29^ It is also well documented that there are greater barriers to accessing physician-based mental health care in rural settings, which may be contributing to the lower likelihood of children and youth living in rural areas using a high intensity of physician-based services.^26^ Our findings are consistent with evidence from adult populations in a similar setting.^27^ Importantly, our findings offer new insight into the relationship between urbanicity and intensity of physician-based mental health service use among children and youth. Specifically, while prior evidence in Ontario found no difference in the six-month prevalence of *any* mental health-related service contact, inclusive of non-physician services, between children and youth living in rural and urban settings, our findings suggest there are meaningful differences between children and youth living in rural/urban settings when assessing cost of mental health-related physician-based service use.^10^ Our findings align with recent evidence from health administrative data in Ontario, suggesting that most children and youth with high mental health care costs live in urban settings.^18^ An important contributor to this result may be the availability and ease of access to mental health related services across rural/urban settings.^30^ Interestingly, we found urbanicity was not significantly associated with being in the top fifth percentile of cost distributions. While the smaller sample sizes of these secondary analyses may be limiting the power to observe association, this finding may also reflect a real-world threshold related to severity of mental disorders and access to high-cost services, where regardless of ease of access to services, at a certain threshold of mental health severity or related impairment, a high-intensity of physician-based services are accessed.

Interestingly, our findings suggest that having more severe symptoms of mental disorders was not significantly associated with high intensity physician-based service use among youth ages 14–17 years. We did, however, find other clinical indicators of significance, specifically prior 12-month suicide attempt, to be associated with high-intensity physician service use. We believe there is a need to continue to study the heterogeneity of mental health related service needs among children and youth in our population to quantify health inequities to guide the design and implementation of policies and programs aimed at reducing inequities over time. Specifically, there is a need to better understand the health trajectories of youth who report prior suicide attempt, such as what services these youth are accessing, inclusive of non-physician services, and whether their mental health needs are being met with currently available services and programs.

### Limitations

There are several notable limitations of our work, including that our findings may have limited generalizability outside of Ontario due to the province-specific approaches to diagnostic coding and mental health service provisions.^31^ We were unable to obtain data from the Ontario Mental Health Reporting System (OMHRS), which contains data on all psychiatric admissions to designated adult psychiatry beds in Ontario for people 16-years and older. It's important to note that although psychiatric hospitalizations to these designated adult beds are not captured in the DAD database, we would have identified children and youth who were admitted to a non-psychiatry bed for mental health reasons, or who were admitted to a child and adolescent psychiatry bed. Starting in 2016, there were two hospital facilities in Ontario that began sending data on youth psychiatric hospitalizations directly to OMHRS, and while we are unable to obtain this information, we believe the impact of this data gap on our findings will be minimal given the small overlap between the reporting shift and our observation window. Recent evidence suggests that approximately one in four adolescent (ages 12–17) psychiatric admissions in Ontario occur in adult psychiatric units.^32^ As such, our findings will underestimate costs associated with psychiatric hospitalizations.

Another important consideration is that our socio-demographic and clinical factors are subject to various biases, including multiple forms of information bias, including recall bias, telescoping bias and selection bias from the potential of differences among participants with, versus without, complete data, all of which may impact our findings.^33–36^ Furthermore, the sampling frame did not include children and youth in the child welfare or youth justice systems, those who are homeless, or Indigenous children living on reserve, who may all be at a greater risk for mental health-related concerns. Another limitation is that due to our small sample sizes, particularly among the sub-sample of older youth ages 14–17, we lacked the power to produce precise estimates. As such, the interpretation of our findings requires caution and future replication.

## Conclusion

Our findings suggest older age, non-rural residency, mental health symptom severity and suicidal behaviour are important factors associated with high-intensity physician-based mental health service use. Quantifying the heterogeneity of mental health-related service needs is a critical component of understanding health inequities in our young population and designing policies to reduce those inequities over time. Future work should aim to continue filling important evidence gaps by better understanding service use trajectories of our young population, inclusive of non-physician service contacts and disentangling frequency, setting and reason for presentation among children and youth with varying intensity of service use over time. This work represents one of the first studies to identify factors associated with high versus low intensity of mental health service use among children and youth. Our findings will support future efforts aimed at better identifying children and youth who may benefit from early and personalized intervention.

## Supplemental Material

sj-docx-1-cpa-10.1177_07067437241300961 - Supplemental material for High Intensity Physician-Based Service Use for Mental Health Concerns in a General-Population Sample of Children and Youth: Utilisation des services de haute intensité dispensés par des médecins pour les problèmes de santé mentale dans un échantillon d’enfants et de jeunes de la population générale Supplemental material, sj-docx-1-cpa-10.1177_07067437241300961 for High Intensity Physician-Based Service Use for Mental Health Concerns in a General-Population Sample of Children and Youth: Utilisation des services de haute intensité dispensés par des médecins pour les problèmes de santé mentale dans un échantillon d’enfants et de jeunes de la population générale by Jordan Edwards, Li Wang, Anne E. Fuller, Kelly K. Anderson, Claire de Oliveira and Katholiki Georgiades in The Canadian Journal of Psychiatry

sj-docx-2-cpa-10.1177_07067437241300961 - Supplemental material for High Intensity Physician-Based Service Use for Mental Health Concerns in a General-Population Sample of Children and Youth: Utilisation des services de haute intensité dispensés par des médecins pour les problèmes de santé mentale dans un échantillon d’enfants et de jeunes de la population générale Supplemental material, sj-docx-2-cpa-10.1177_07067437241300961 for High Intensity Physician-Based Service Use for Mental Health Concerns in a General-Population Sample of Children and Youth: Utilisation des services de haute intensité dispensés par des médecins pour les problèmes de santé mentale dans un échantillon d’enfants et de jeunes de la population générale by Jordan Edwards, Li Wang, Anne E. Fuller, Kelly K. Anderson, Claire de Oliveira and Katholiki Georgiades in The Canadian Journal of Psychiatry
