# Supplementary material for: High Intensity Physician-Based Service Use for Mental Health Concerns in a General-Population Sample of Children and Youth: Utilisation des services de haute intensité dispensés par des médecins pour les problèmes de santé mentale dans un échantillon d’enfants et de jeunes de la population générale
Source: Can J Psychiatry. 2025 Jan 6;70(2):127–35. doi: 10.1177/07067437241300961 (PMC11733866; doi:10.1177/07067437241300961)
Supplement: sj-docx-1-cpa-10.1177_07067437241300961 - Supplemental material for High Intensity Physician-Based Service Use for Mental Health Concerns in a General-Population Sample of Children and Youth: Utilisation des services de haute intensité dispensés par des médecins pour les problèmes de santé mentale d [file sj-docx-1-cpa-10.1177_07067437241300961.docx]

**Supplement 1:** Data Dictionary.

| Category | Definition | Data Source/Provider | Codes/Questions |
| --- | --- | --- | --- |
| Outcome / Exposure | | | |
| Mental Health Service Codes used to determine costs. | Any mental health related visit in the 24-month period post OCHS. | Ontario Health Insurance Program (OHIP)  National Ambulatory Reporting System (NACRS)  Discharge Abstract Database (DAD) | *Any mental health related diagnosis codes:*   - **F10-19, F55** Substance-related disorders - **F20 (excluding F20.4), F22-25, F28, F29, F53.1** Schizophrenia - **F21, F60-62, F68, F69, F80-F84, F88-92, F94, F95, F98** Neurodevelopmental and personality disorders - **F30-F39**: Mood disorders - **F40-48**: Anxiety disorders - **F50.0-50.3, F50.8, F50.9** Eating disorders - **F90** Hyperkinetic disorders - **F92** Mixed disorders of conduct and emotions - **F93** Emotional disorders with onset specific to childhood - **F98.8** Other specified behavioural and emotional disorders with onset usually occurring in childhood and adolescence - **F98.9** Unspecified behavioural and emotional disorders with onset usually occurring in childhood and adolescence - **F99** Mental disorder, not otherwise specified - **291, 292, 299** Alcohol, drug or other psychoses - **295-298** Psychotic disorders - **300** Neurotic disorders (includes reactive depression) - **301, 302, 306, 309** Non-psychotic disorders - **303, 304** Substance use disorders - **307** Habit spasms, tics, anorexia nervosa, sleep disorders - **311** Depressive disorder, not elsewhere classified (Include: depressive disorder NOS, depressive state NOS, depression NOS) - **313** Disturbance of emotions specific to childhood and adolescence - **314** Hyperkinetic syndrome of childhood - **315** Developmental delay - **316** Psychic factors associated with diseases classified elsewhere - **897-902, 904-906, 909** Social problems |
| Clinical / Socio-demographical Factors | | | |
| Clinical Factors | Factors related to individual-level mental health needs in the period surrounding completion of the OCHS survey. | OCHS survey.  Questions asked to either person most knowleable (PMK) or self. | - PMK - Mental health severity: Total score from the OCHS dimensional scales (past 6-months). - PMK – Mental Health Impairment: Total score from the follow-up module to the OCHS-EBS. If people endorsed any mental health challenge, a series of follow-up questions were asked about whether these challenges have interfered with daily life functioning (e.g. making and keeping friends, learning or class work). The four questions use a five point likert scale ranging from not at all to a great deal. - *Self - Substance use: any tobacco use, cannabis or illicit drug use, heavy episodic drinking (past 6-months). - *Self - Any suicidal ideation, suicidal attempt, non-suicidal self-injury (past 12-months). - *Self - Any child maltreatment: youth experience of any physical, sexual, exposure to intimate partner violence, emotional abuse or neglect (lifetime). - *Self - Problematic eating: Total score from the eating behaviours scales, which includes questions related to anorexia and bulimia (past month). The seven questions in the module (e.g. how often did you avoid eating or refuse to ear) use a five point likert scale, which ranges from never to almost every day. - PMK - Any childhood chronic condition: diagnosed by a health professional with any of the following long-term conditions for diabetes, epilepsy, cerebral palsy, kidney condition or disease, ecxema, any other long-term condition (lifetime).   - We’ve excluded conditions: bronchitis, food or digestive allergies, respiratory allergies such as hay fever, any other allergies, asthma. - PMK self – Parental Distress - Psychological distress measured using the Kessler 10 (past 1-month). - PMK self – Parental MH Diagnosis - Any prior mental health diagnosis from a doctor or counselor (lifetime). - Prior Service Use (Below) |
| Prior Service Use | Any mental health related service visit in the 6-month period pre-OCHS survey. | Non-physician service visit obtained through parental-report or youth self-report from OCHS survey.  Physician visits obtained from OHIP, emergency visits obtained from NACRS, and hospitalizations obtained from DAD. | *Non-physician services, endorsing any of the following questions regarding service visit over the past 6-months:*   - Seen a care provider about mental health concerns-Psychologist - Seen a care provider about mental health concerns-Social worker - Seen a care provider about mental health concerns-Other type of counsellor - Seen a care provider about mental health concerns-School guidance counsellor - Other help-teacher or other adult at school - School individual or group counselling - Ministry of Child and Youth Services (MCYS) mental health agency visit for mental health concerns.   *Physician services:*  Obtained using diagnostic codes from OHIP, NACRS, and DAD, described above. |
| Socio-demographic Factors | Other contextual factors captured in the period surrounding the completion of the OCHS survey. | OCHS survey  Questions asked to PMK or derived from census. | - Age - Sex (0=female; 1=male) - Parental immigrant status (0=both parents born in Canada; 1=at least 1 foreign born parent) - Number of biological parents in the home (0=two biological parents; 1=one or no biological parents) - Household income (0=not poor; 1=poor). This indicator was based on the 2013 before tax-cut-offs in Canada. - Rurality: Calculated based on population size and density, urban-rural residency is a binary indicator where those living in rural residences (<1000 in a region or <400 residents per km^2^) |
| *14-17 age group. | | | |
